# Early Gabapentin Treatment during the Latency Period Increases Convulsive Threshold, Reduces Microglial Activation and Macrophage Infiltration in the Lithium-Pilocarpine Model of Epilepsy

**DOI:** 10.3390/ph10040093

**Published:** 2017-11-28

**Authors:** Alicia Rossi, Veronica Murta, Jerónimo Auzmendi, Alberto Javier Ramos

**Affiliations:** 1 Departamento de Histología, Embriología, Biología Celular y Genética, Facultad de Medicina, Universidad de Buenos Aires, Buenos Aires CP1121, Argentina; ivanhoe_rowena@hotmail.com; 2 Laboratorio de Neuropatología Molecular, Instituto de Biología Celular y Neurociencia “Profesor E. De Robertis” IBCN UBA-CONICET, Buenos Aires CP1121, Argentina; vmurta.fmed@gmail.com (V.M.); jeronimo.auzmendi@gmail.com (J.A.)

**Keywords:** epilepsy, latency period, reactive gliosis, synaptogenesis, epileptogenesis

## Abstract

The lithium-pilocarpine model of epilepsy reproduces several features of temporal lobe epilepsy in humans, including the chronological timeline of an initial latency period followed by the development of spontaneous seizures. Epilepsy therapies in humans are implemented, as a rule, after the onset of the spontaneous seizures. We here studied the potential effect on epileptogenesis of starting an early treatment during the latency period, in order to prevent the development of spontaneous seizures. Adult male Wistar rats were treated with 3 mEq/kg LiCl, and 20 h later 30 mg/kg pilocarpine. Once status epilepticus (SE) was achieved, it was allowed to last for 20 min, and then motor seizures were controlled with the administration of 20 mg/kg diazepam. At 1DPSE (DPSE, days post-status epilepticus), animals started to receive 400 mg/kg/day gabapentin or saline for 4 days. At 5DPSE, we observed that SE induced an early profuse microglial and astroglial reactivity, increased synaptogenic trombospondin-1 expression and reduced AQP4 expression in astroglial ending feet. Blood brain barrier (BBB) integrity seemed to be compromised, as infiltrating NG2+ macrophages and facilitated access to the CNS was observed by transplanting eGFP+ blood cells and bone marrow-derived progenitors in the SE animals. The early 4-day gabapentin treatment successfully reduced microglial cell reactivity and blood-borne cell infiltration, without significantly altering the mRNA of proinflammatory cytokines IL-1β and TNFα immediately after the treatment. After 21DSPE, another group of animals that developed SE and received 4 days of gabapentin treatment, were re-exposed to subconvulsive accumulative doses of pilocarpine (10 mg/kg/30 min) and were followed by recording the Racine scale reached. Early 4-day gabapentin treatment reduced the Racine scale reached by the animals, reduced animal mortality, and reduced the number of animals that achieved SE (34% vs. 72%). We conclude that early gabapentin treatment following SE, during the latency period, is able to reduce neuroinflammation and produces a persistent effect that limits seizures and increases convulsive threshold, probably by restricting microglial reactivity and spurious synaptogenesis.

## 1. Introduction

Epilepsy is a prevalent neurological disorder worldwide. It affects nearly 1% of the world population [1]; more than 65 million patients. The epileptic state is characterized by recurrent spontaneous abnormal electrical discharges in the brain due to an altered excitatory/inhibitory (E/I) neurotransmission balance. The resulting seizures alter patients’ perception, consciousness, memory and motor activity. There are a significant number of antiepileptic drugs (AED) on the market, however these therapeutic agents are only aimed at controlling the seizures; there are no disease-modifying therapies available (reviewed in [2]). In other words, the AED target only the disease symptoms, and fail to adequately prevent seizure development, or permanently halt the occurrence of seizures [2]. On the other hand, approximately 30% of epileptic patients immediately present or will present after years of AED treatment resistance to the treatment, showing intractable seizures that do not respond to AED or combinations of drugs. For these patients, invasive approaches like surgical ablation of the epileptic foci or vagus nerve stimulation are the unique therapeutic options [3,4].

Retrospective studies performed on temporal lobe epilepsy (TLE) patients, the most prevalent epilepsy in humans, have shown that a significant number of patients refer an early event of brain injury during childhood. Cumulative clinical and experimental data have raised the hypothesis that early brain injury is an initial precipitating event (IPE) that triggers a cascade of molecular and cellular consequences leading to epilepsy (reviewed in [5]). The period following this early event is called the latency period and it may last several years in humans (2–15 years). A similar disease progression can be mimicked in animal models in a shorter period of time, by inducing an IPE with pro-convulsive drugs that is followed by a latency period and the subsequent development of chronic seizures. Given that most studies have been conducted once the chronic phase had been reached, there is relatively scarce information about the latency period, which would probably offer several key elements to better understand the epileptogenic process, and would allow the identification of novel therapeutic targets that could achieve disease-modifying effects. The precise mechanisms as well as the temporal sequence of events during the latency period are not clearly defined, however the prevailing hypothesis in the field is that epileptogenesis involves several remodeling events induced by neuroinflammation and neuronal apoptosis, such as exacerbated synaptic plasticity, synaptogenesis and circuit remodeling, which lead to an altered E/I balance, hyperexcitability and spontaneous seizures (reviewed in [2]). The findings in human surgically resected hippocampi of epileptic patients have shown several of these alterations, including loss of projecting neurons and interneurons, neurogenesis, mossy fibers sprouting with aberrant reconnection, neuroinflammation, reactive gliosis and altered blood-brain barrier (BBB) permeability (reviewed in [6]).

Clinical and experimental evidence have shown that the innate immunity and subsequent activation of inflammatory pathways contribute to the development of epilepsy [7,8]. Astrocytes and microglia are the innate immunity effectors in the brain and play a major neuroinflammatory role in Central Nervous System (CNS) diseases, including epilepsy. In particular, reactive astrocytes are able to secrete molecules that have pro-synaptogenic effects (reviewed in [9]) that may support spurious synaptogenesis occurring during the latency period of epilepsy [10,11,12,13,14]. Available data have shown that epilepsy is related to inflammation with increased levels of cytokines, and studies performed on epileptic patients demonstrated that cytokine level correlates with the intensity and duration of epileptic seizures [15], while experimental models of epilepsy have shown that microglia, brain resident macrophages and dendritic cells also become activated [16].

Thrombospondin 1 (TSP-1) is an astroglial-derived synaptogenic molecule that showed the ability to induce excitatory synapses [17] by interacting with the α2δ1 subunit of voltage-sensitive calcium channels [18,19]. TSP-1 expression is augmented in astrocytes following brain injury and seizures [20] and mice overexpressing the α2δ1 subunit show epileptiform activity [11]. On the other hand, gabapentin, an antagonist of TSP-1 receptor α2δ1, administered after brain cortex trauma reduces the incidence of epileptiform discharges and has neuroprotective effects probably by suppressing the excitatory synapse formation [9,21]. Although originally developed as a gabaergic agonist, gabapentin showed low effectiveness as AED. Today, gabapentin’s main therapeutic use is to treat neuropathic pain where its effect seems to rely on the reduction of reactive microgliosis [22]. In addition, we have previously shown that subacute gabapentin administration during the latency period, that follows the IPE in the pilocarpine model of TLE, reduces neuronal death, increases neuronal plasticity, and diminishes reactive gliosis and neuroinflammation in the long term [23]. Here we show that gabapentin treatment during an early therapeutic window following IPE reduces glial activation and macrophage infiltration in the pilocarpine model of TLE. These early effects are long-lasting, as shown by the increased epileptic threshold in animals re-exposed to sub-convulsive pilocarpine doses. Our results show that controlling reactive gliosis in an early time-window following IPE is a suitable disease-modifying strategy in epilepsy.

## 2. Results

### 2.1. Higher Expression of Innate Immunity Mediators during the Latency Period after SE

**Figure 1:**
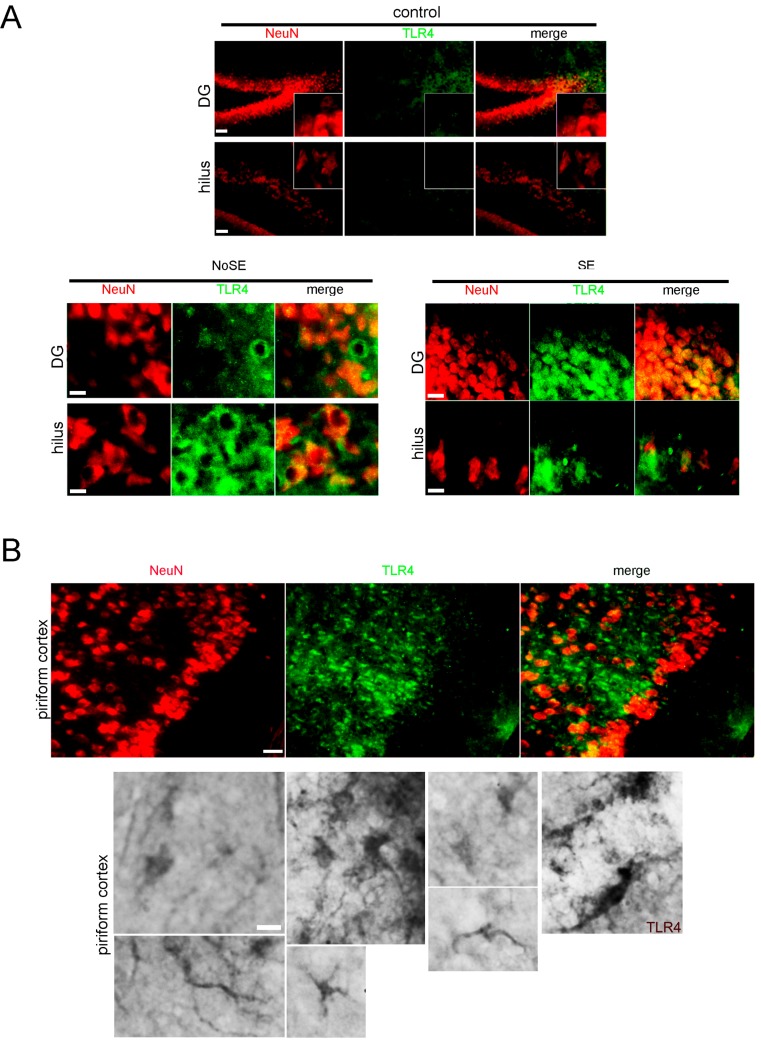
TLR4 expression in the lithium-pilocarpine experimental model of temporal lobe epilepsy (TLE). (**A**) Representative photomicrograph of hippocampal brain tissue sections immunostained for TLR4 (green) and neurons were identified with the NeuN neuronal specific marker (red). No-SE animals were those that reached Racine 1–2, without reaching sustained Racine 3–5 that was considered as status epilepticus (SE). Images were obtained in the hippocampal hilus and dentate gyrus (DG) at 15 days after pilocarpine-induced seizures. Scale bars: 30 μm (control panel); 15 and 10 μm (No-SE panel); 30 μm and 15 μm (SE panel). (**B**) **Upper panel**: Representative photomicrograph of piriform cortex brain tissue sections immunostained for TLR4 (green) showing small rounded cells that invaded the piriform cortex that lost NeuN neurons (red) at 21DPSE. Scale bar: 30 μm. **Lower panel**: Immunohistochemistry for TLR4 showing the expression in glial cells and neuronal projections from piriform cortex at 21 days post SE (21DPSE). Scale bar: 10 μm.

**Figure 2:**
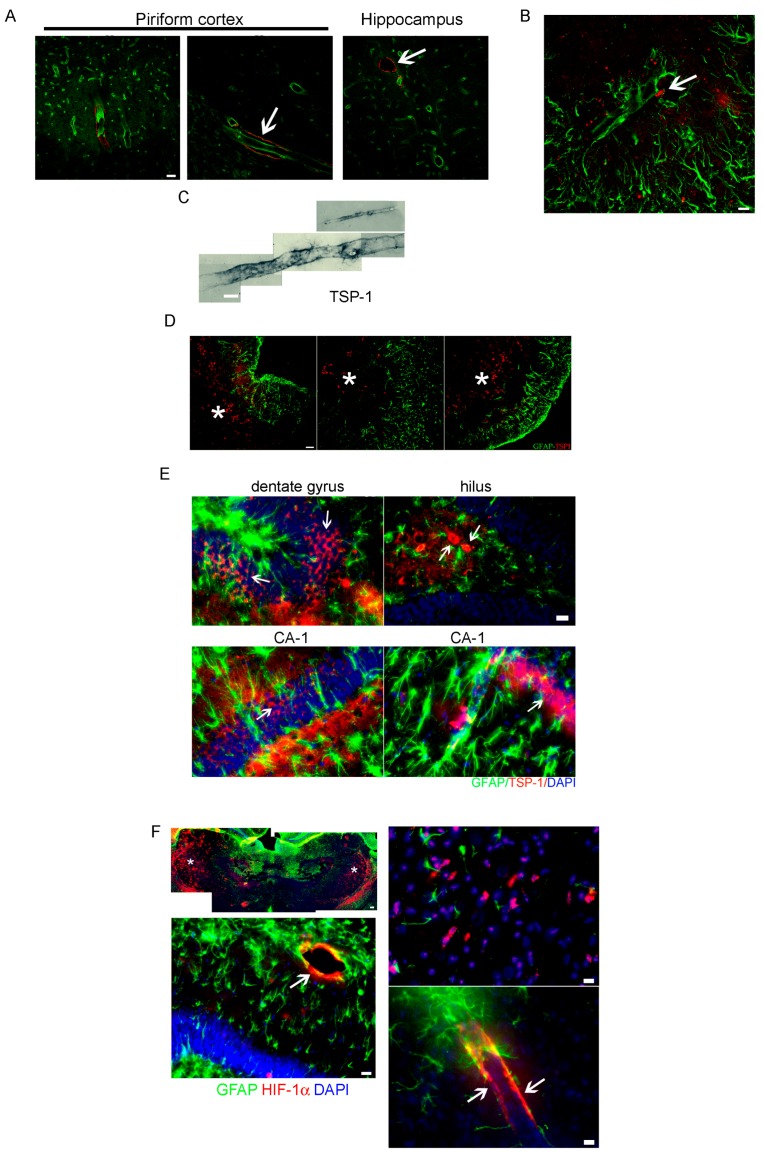
Synaptogenic TSP-1 is expressed after status epilepticus (SE). (**A**) Confocal images of TSP-1 expression (red) surrounding in brain blood vessels (arrows) at 3DPSE. Blood vessels were identified with Tomato Lectin-FICT (green). Scale bar: 30 μm. (**B**) Infiltrating macrophages expressing TSP-1 (red) on the blood vessels endothelium (arrow) and astroglial end-feet evidenced by the astroglial Glial Fibrillary Acidic Protein (GFAP) immunostaining (green) at 7DPSE. Scale bar: 30 μm. (**C**) Photocomposition of a blood vessel and astrocytes immunostained for TSP-1 and revealed by diaminobenzidine (DAB)—developed immunohistochemistry at 7DPSE. Scale bar: 10 μm. (**D**) Confocal low magnification image showing TSP-1 (red) localization in macrophages infiltrating the piriform cortex (asterisk) at 7DPSE where astrocytes GFAP+ (green) disappeared after the SE. Scale bar: 45 μm. (**E**) The panel shows different hippocampal areas immunostained for GFAP (green) and TSP-1 (red) where perivascular astrocytes and neuronal somata (arrows) are also stained for TSP-1. Scale bar: 15 μm. (**F**) The panel presents several images of HIF-1α immunostaining (red, arrows) in the areas where TSP-1 overexpression was detected. Overlapping in merged pictures is seen in yellow. Scale bars: 50 μm (upper left image); 30 μm (lower left image); 15 μm (right images).

Following pilocarpine administration, the development of epileptic seizures was behaviorally monitored as previously described [23]. Seizures had their onset 20–40 min after pilocarpine injection and duration was limited to 20 min by diazepam administration. Animals that developed seizures were classified according to the Racine scale [24,25]. Sustained Racine stages 3–5, without returning to normal behavior, was considered as status epilepticus (SE) [23,26]. We have previously characterized the neuronal degeneration and reactive gliosis observed during the latency period that follows SE [23] and described an ischemic-like core lesion in the piriform cortex also reported by other groups [26,27]. Here, we first analyzed the expression of a number of key molecules that could be related to the spurious synaptogenesis induced by glial-derived substances. As shown in Figure 1A, we observed that the innate immunity-related Toll-like-4 (TLR4) receptor was expressed in the hippocampus and piriform cortex in microglia and neurons that show atypical NeuN cytoplasmic staining at 15DPSE (DPSE, days post-status epilepticus). As shown in Figure 1A, TLR4 expression was more intense in animals that reached higher Racine index. Then, at 21 DPSE, TLR4 immunostaining was detected in macrophages and microglia infiltrating the piriform cortex and astrocytes (Figure 1B). It has been described that reactive glia may re-express synaptogenic molecules that were silenced after brain development (reviewed in [9,28]). Having this in mind, we studied the TSP-1 expression during the latency period after SE. Figure 2A shows that TSP-1 expression was detected in the perivascular astrocytes at 3DPSE, a sticking contrast with the weak and diffuse staining in control brains (Supplementary Figure S1). Then, at 7DPSE, TSP-1 immunostaining was present in macrophages that infiltrate the ischemic-like lesion core in the piriform cortex (Figure 2B–D). Detailed images of the dentate gyrus and hippocampal CA-1 neurons also show TSP-1 immunostaining in neuronal soma, probably due to TSP-1 binding to the α2δ1 subunit of the voltage-sensitive calcium channels (Figure 2E). TSP-1 expression was shown to be controlled by different transcription factors, including Hypoxia-Induced Factor 1 alpha (HIF-1α) [29,30]. As shown in Figure 2F, HIF-1α immunostaining was observed at early time points (3DPSE) during the latency period. This expression was detected in the endothelium of striatal and hippocampal blood vessels. We conclude that innate immunity receptor TLR4 and synaptogenic TSP-1 are expressed in the early latency period, the latter probably due to the temporary hypoxic episodes during the seizures in the SE.

### 2.2. Altered Blood Brain Barrier (BBB) Permeability during the Latency Period after SE

**Figure 3:**
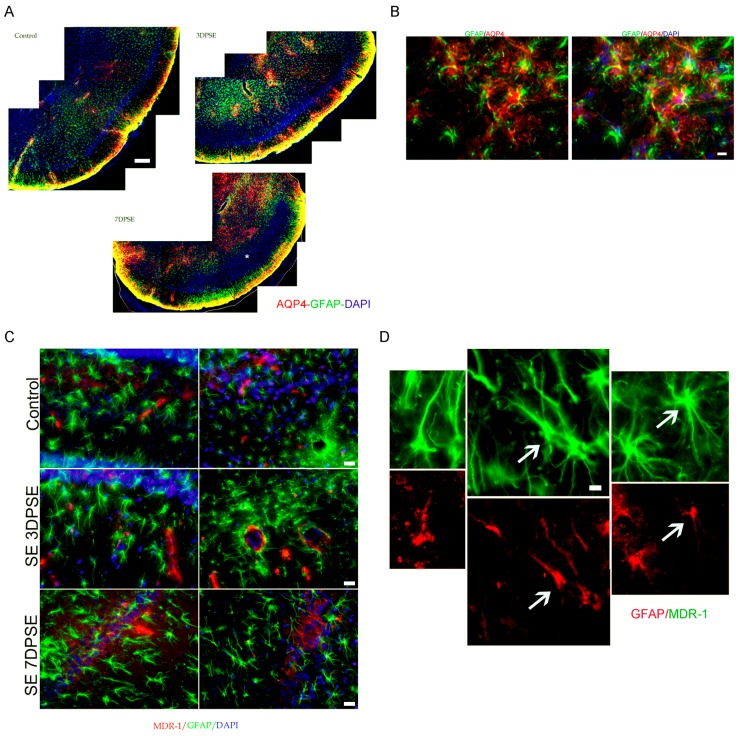
Altered expression of blood vessels permeability after SE. (**A**) Representative images of the alteration in AQP4 expression (red) in the piriform cortex of 3DPSE and 7DPSE where GFAP+ astrocytes (green) were also reduced (asterisk) and yellow represents GFAP/AQP4 overlapping. Scale bar: 500 μm. (**B**) High magnification details of AQP4 relocalization in astrocytes from the end-feet to the astroglial soma at 7DPSE in the piriform cortex. Scale bar: 15 μm (**C**) Pgp-170 (MDR-1) expression is induced in blood vessels endothelium and astrocytes from hippocampus at 3 and 7DPSE. Scale bar: 20 μm. (**D**) High magnification image showing the coexpression of GFAP (green) and Pgp-170 (MDR-1) (red) in the hippocampus at 7DPSE (arrows). Scale bar: 8 μm.

Altered BBB permeability has been documented in experimental epilepsy models and human surgically resected epileptic tissue [31,32,33]. BBB alteration is also induced by the IPE and thus it is thought to be involved in the epileptogenic process [31,34]. We here studied the astroglial aquaporin-4 (AQP4), a protein highly expressed in the astroglial end-feet forming an essential part of the BBB. As shown in Figure 3A, AQP4 expression was altered by the SE showing a reduced expression in the piriform cortex and hippocampus during the latency period at 7DPSE. In addition, AQP4 showed relocalization from astrocytic end-feet to glial soma (Figure 2B). Pgp-170 (MDR-1) is a BBB efflux transporter that is regarded as one of the factors inducing AED resistance in patients [35,36]. In agreement with the recent report of Hartz and colleagues [36], we observed that Pgp-170 was overexpressed after the pilocarpine-induced SE in the blood vessels by 3DPSE (Figure 3C). Then, at 7DPSE, Pgp-170 overexpression persisted in astrocytes and blood vessel endothelium (Figure 3C,D). We conclude that key molecules involved in the transport through the BBB are altered by the SE. 

**Figure 4:**
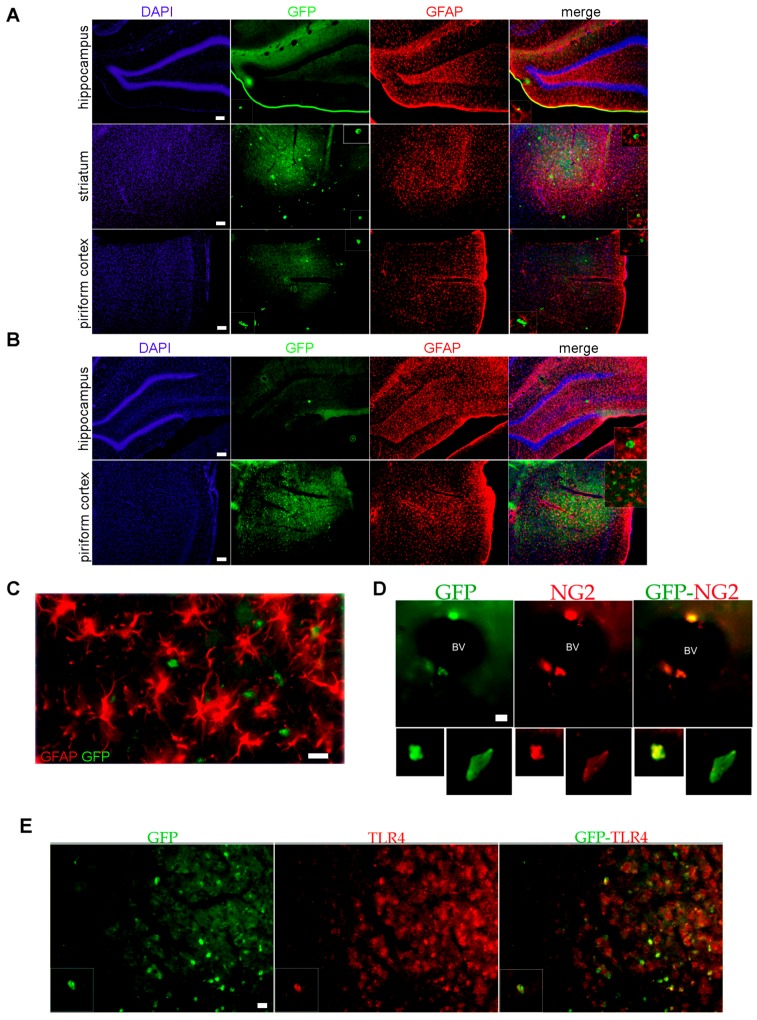

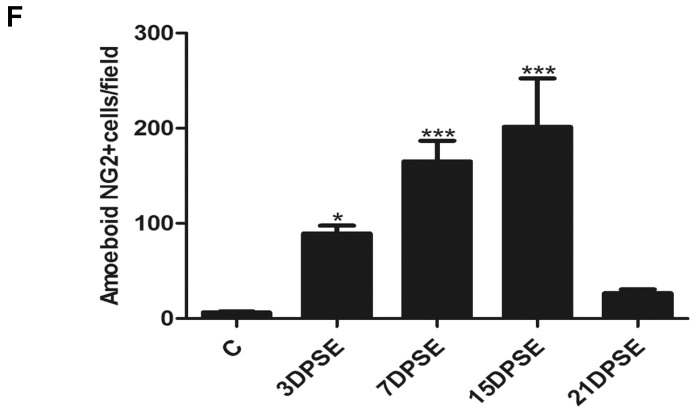
Increased permeability of blood brain barrier (BBB) to blood-borne cells after SE. (**A**) Representative images of eGFP+ leukocytes from donator transgenic Wistar-TgN(CAG-GFP)184ys rats (green), infiltrating the brain of SE wild type rats after 3DPSE; counter staining was made with GFAP (red) and 4′,6-diamidino-2-phenylindole (DAPI) nuclear staining (blue). Scale bar: 30 μm. (**B**) A similar experiment, but injecting bone marrow myeloid cells (BMMC) obtained from eGFP+ rats. Scale bar: 30 μm. (**C**) Higher magnification detail showing the eGFP+ leukocytes infiltrating the piriform cortex colocalizing with GFAP+ astrocytes (red). Scale bar: 30 μm. (**D**) Images showing some eGFP+ cells adhered to the endothelium of brain blood vessels and immunostained for NG2 macrophage marker (red) at 3DPSE. Yellow color represents overlapping of both markers. Scale bar: 10 μm. (**E**) Representative images showing that infiltrating eGFP+ macrophages (green) are also immunostained for TLR4 (red); images were obtained in the piriform cortex at 3DPSE. Scale bar: 30 μm. (**F**) Quantification of NG2+ amoeboid infiltrating macrophages in a wild type rat at different times after SE. Significance was evaluated by one-way ANOVA and Bonferroni post-test, data are expressed as mean ± SEM. (*) *p* < 0.05; (***) *p* < 0.001.

Altered BBB cell permeability may have also consequences on the innate immunity response. Not only does serum albumin behave as a damage associated molecular pattern (DAMP) activating innate immunity local response and reactive gliosis in the CNS, but also blood-derived leukocytes access to the CNS is facilitated after BBB disruption [37,38]. To test BBB cell permeability during the latency period, we transferred at 1DPSE peripheral leukocytes or bone marrow myeloid cells (BMMC) from the transgenic eGFP+ rat strain [Wistar-TgN(CAG-GFP)184ys] to animals that developed SE. As shown in Figure 4A,B, eGFP+ leukocytes or BMMC were observed infiltrating the hippocampus, striatum and piriform cortex by 3DPSE. Infiltrating eGFP+ cells colocalized with reactive astrocytes in the affected areas at 3DPSE (Figure 4C) and were also observed in the endothelial layer of blood vessels (Figure 4D). Infiltrating eGFP cells also showed TLR4 expression typical of the myeloid-engaged lineage (Figure 4E). In agreement with these results, increased abundance of NG2+ blood-derived macrophages were observed in the hippocampus, striatum and piriform cortex at 3DPSE (Figure 4F).

### 2.3. Early Gabapentin Treatment in the Latency Period Reduces Reactive Gliosis

**Figure 5:**
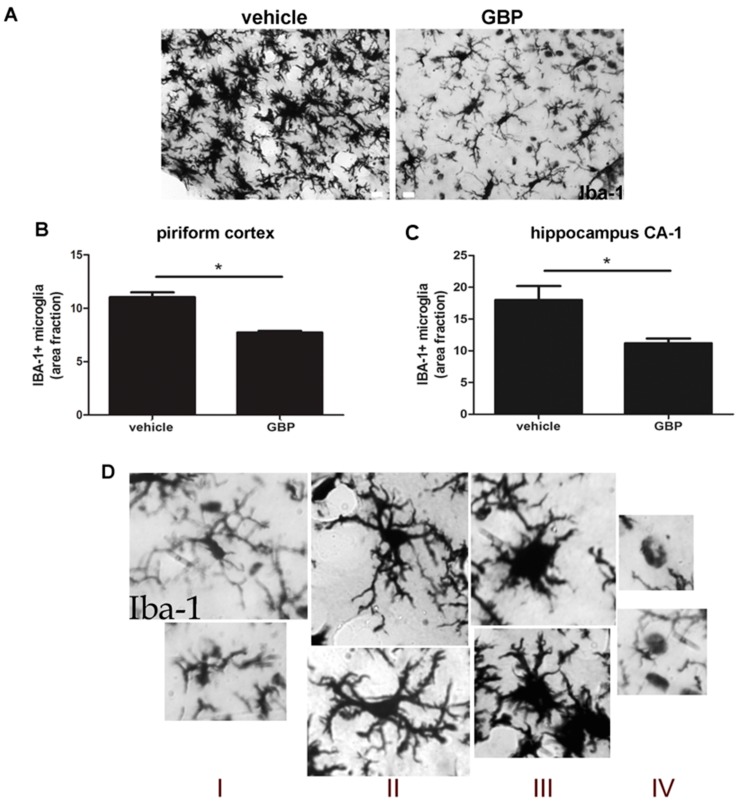

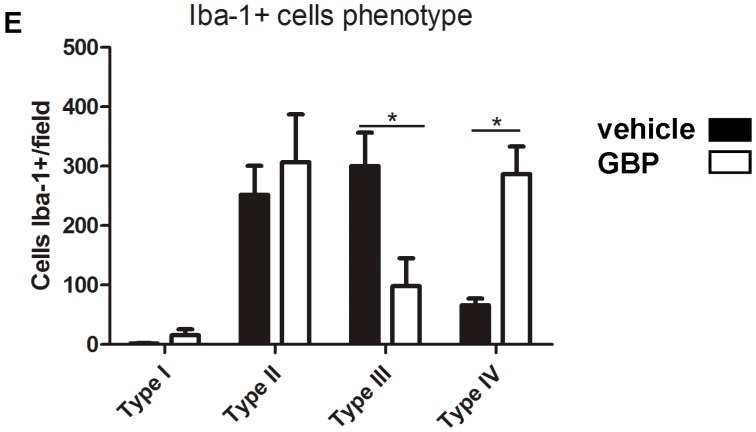
Gabapentin treatment reduces reactive microgliosis in SE animals. (**A**) Representative images of Iba-1+ immunostained microglial cells in the piriform cortex of gabapentin- (GBP) and vehicle-treated animals at 5DPSE. Scale bar: 15 μm. (**B**,**C**) Quantitative analysis of area fraction occupied by Iba-1+ microglial cells in GBP- and vehicle-treated animals after 5DPSE in the piriform cortex and hippocampal stratum radiatum CA-1 respectively. Significance was evaluated by Student-*t*-test (* *p* < 0.05), data are expressed as mean ± SEM. (**D**) Representative images of microglial phenotypes used for characterizing the reactive microgliosis in type I, II, III and IV. (**E**) Quantitative analysis of the Iba-1+ microglial phenotypes in the piriform cortex after 5DPSE in vehicle- (white bars) and GBP-treated (black bars) animals. Data are expressed as mean ± SEM; (* *p* < 0.05) after one-way ANOVA and Bonferroni post-test.

We have previously shown that SE induced a long-lasting reactive gliosis and that gabapentin chronic treatment was able to reduce reactive gliosis by 21DPSE [23]. However, it was not clear if gabapentin treatment was directly limiting reactive gliosis in a specific time-window after the IPE, or if the effect was secondary to the improvement in neuronal survival also produced by the gabapentin treatment. In order to answer this question, we here analyzed the reactive gliosis pattern immediately after a short 4-day gabapentin treatment administered after SE. As shown in Figure 5A–C, gabapentin treatment was able to significantly reduce microglial activation when analyzed by Iba-1 immunostaining. In addition, the detailed analysis of microglial phenotypes (Figure 5D) showed that gabapentin treatment during the 4-day time-window induced a shift in the phenotype abundance towards a microglial phagocytic resolutive phenotype (type IV) and a reduction in the proinflammatory type III phenotype (Figure 5E).

**Figure 6:**
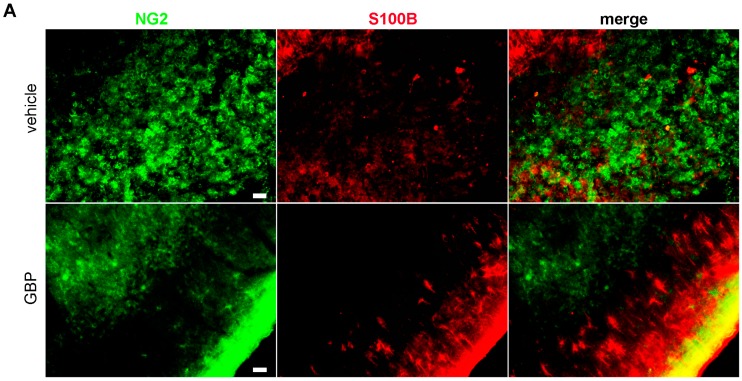

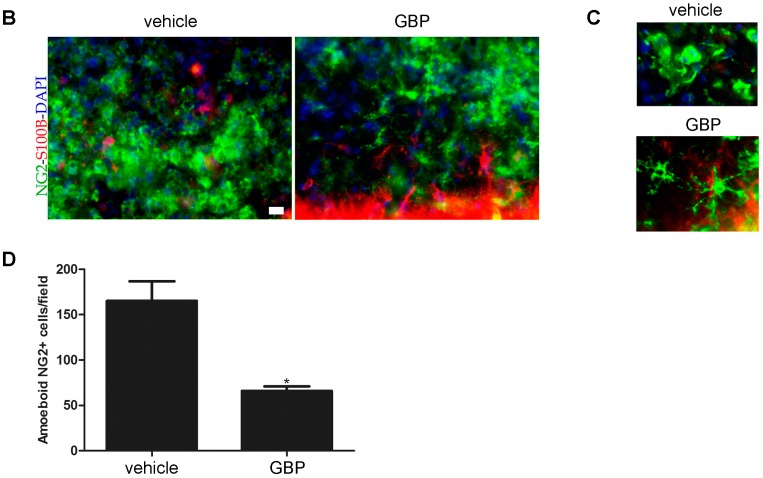
Gabapentin treatment reduces macrophage infiltration in SE animals. (**A**) Representative images showing the reduction in amoeboid NG2-immunoreactive infiltrating macrophages (green) in the piriform cortex of gabapentin (GBP) or vehicle-treated SE animals after 7DPSE. Counter staining with astroglial marker S100B (red) and DAPI nuclear staining (blue) are shown. Yellow color shows overlapping of both markers. Scale bar: 30 μm. (**B**,**C**) Higher magnification images showing the change in NG2+ cells morphology from the macrophage-like amoeboid phenotype in 7DPSE vehicle-treated animals to the typical NG2+ oligodendrocyte precursor (OPC) stellated staining observed in GBP-treated animals. Scale bar: 15 μm. (**D**) Quantitative analysis of amoeboid NG2+ cells in the GBP- and vehicle-treated animals after 7DPSE in the piriform cortex. Significance was evaluated by Student-*t* test (* *p* < 0.05), data are expressed as mean ± SEM.

The NG2 chondroitin sulfate proteoglycan is expressed by CNS resident polydendrocytes (OPC) but also by blood-derived macrophages invading the CNS. While NG2+ resident polydendrocytes are stellated cells uniformly distributed in the CNS parenchyma, invading NG2+ macrophages are amoeboid phagocytic cells. Microscopy observations and quantitative analysis of the NG2+ phenotypes during the latency period showed that the early 4-day gabapentin treatment significantly reduced the NG2+ macrophages with amoeboid phenotype (Figure 6A,B) and increased the OPC-like stellated NG2+ cells, when compared to vehicle treatment by 7DPSE (Figure 6C,D).

**Figure 7:**
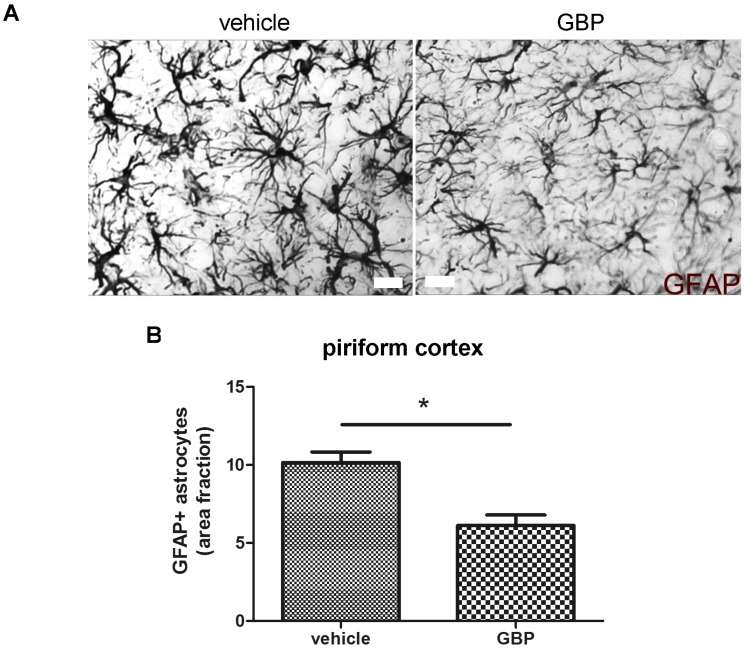

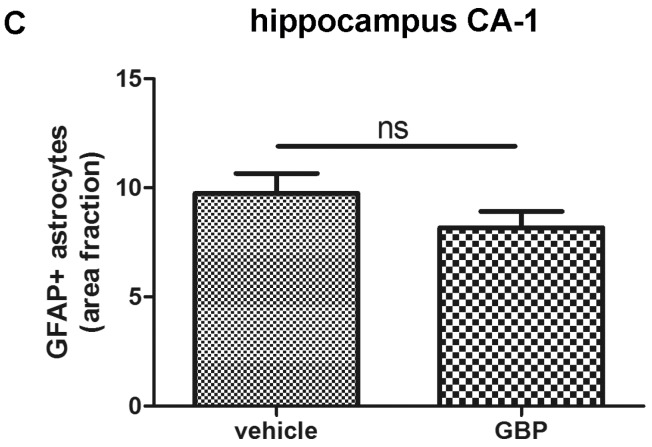
Gabapentin treatment reduced reactive astrogliosis in the piriform cortex of SE animals. (**A**) Representative images of GFAP+ immunostained astrocytes cells in the piriform cortex of gabapentin- (GBP) and vehicle-treated animals at 5DPSE, showing heterogeneous astroglial response to GBP treatment. Scale bar: 15 μm. (**B**) Quantitative analysis of area fraction occupied by GFAP+ astrocytes in the piriform cortex. (**C**) Quantitative analysis of area fraction occupied by GFAP+ astrocytes in the stratum radiatum of hippocampal CA-1. Significance was evaluated by two tailed Student-*t* test; data are expressed as mean ± SEM; (*) represents *p* < 0.05 and n.s. is not statistically significant.

Reactive astrogliosis during the latency period after SE was previously described in detail by us [23]. Here, we studied the reactive astrogliosis after the early 4-day gabapentin treatment and compared it with the vehicle treated SE-exposed animals after 5DPSE. As shown in Figure 7A,B, Glial Fibrillary Acidic Protein (GFAP) immunostained astrocytes showed less hypertrophy evidenced by the reduction in the area ocupied by astrocytes in the piriform cortex. On the other hand, the reduction in astroglial hypertrophy did not reach statistical significance in the hippocampal CA-1 area (Figure 7C). The astrocytes number was not altered by the gabapentin treatment (data not shown).

**Figure 8:**
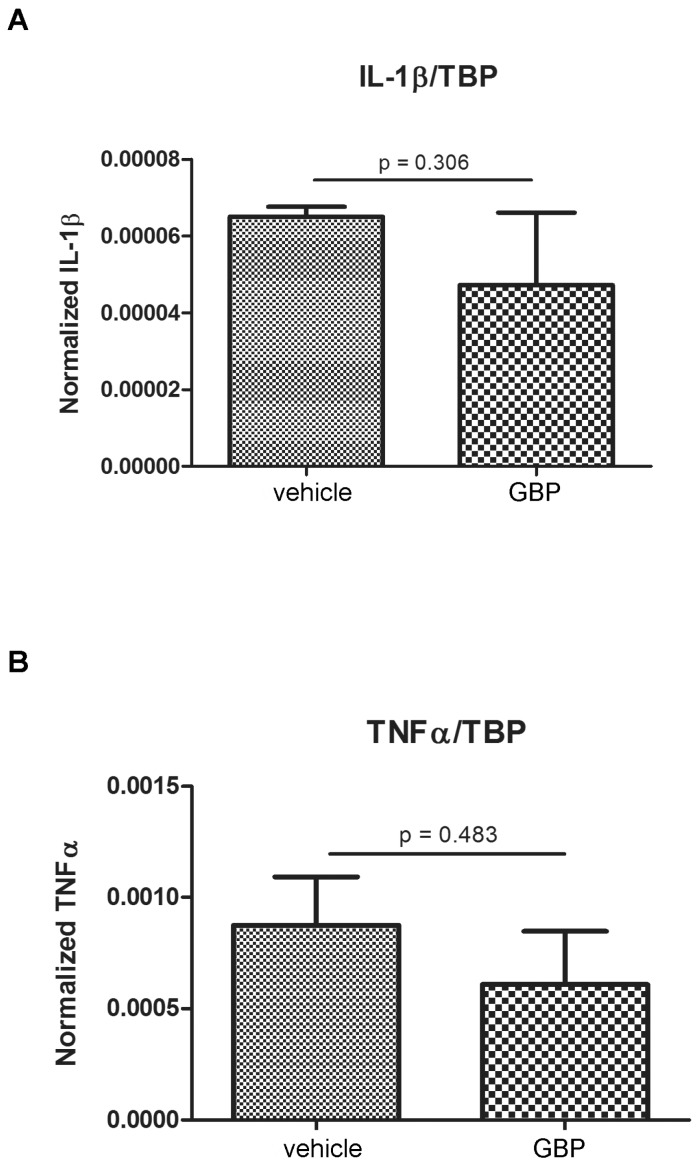
Gabapentin treatment does not significantly reduce reactive cytokine expression in SE animals. (**A**,**B**) Quantitative real time RT-PCR studies of IL-1β and TNFα mRNA expression in the hippocampus of vehicle- or GBP-treated SE animals. Data are relative to the housekeeping gene TBP and are represented as mean ± SEM of 3 determinations. Statistical significance was analyzed using the two tailed Student-*t*-test.

Having in mind that microgliosis and blood-derived macrophage infiltration were limited by the early gabapentin treatment, we then tested if proinflammatory cytokine expression was also altered by the 4-day gabapentin treatment. Figure 8 shows that, surprisingly, gabapentin treatment did not produce a significant change in the IL-1β and TNFα levels by real time-RT Polymerase Chain Reaction (PCR) when comparing gabapentin vs. vehicle-treated animals that developed SE. 

We conclude that early gabapentin treatment reduces microglial reactivity and blood-borne cell infiltration, without producing a sustained change in the expression of the proinflammatory mediators IL-1β and TNFα.

### 2.4. Gabapentin-Treated Animals Have Increased Convulsive Threshold and Increased Survival When Re-Exposed to Convulsive Agent 

**Figure 9:**
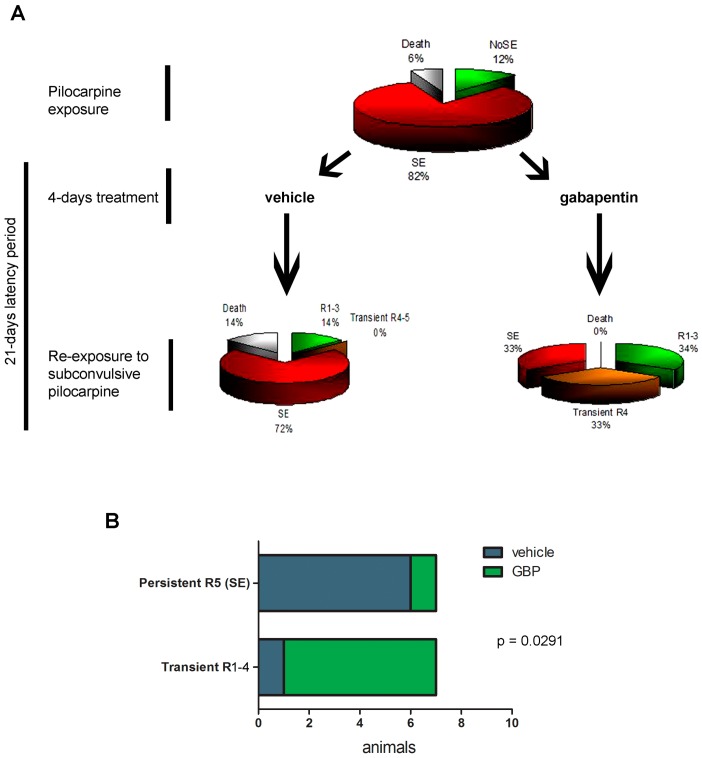
Early 4-day gabapentin treatment increases convulsive threshold to a subsequent pro-convulsive stimulus. (**A**) Schematic representation of the pilocarpine re-exposure showing the distribution of the Racine scale level achieved by the animals. (**B**) Statistical evaluation by Fisher’s exact test of a typical experiment involving in this case 14 animals. Note that most GBP treated animals did not reach SE (sustained Racine 5 level).

Having in mind that early gabapentin treatment during a short time-window after the experimental SE efficiently reduced microglia activation, blood-derived macrophage infiltration was previously shown to reduce neuronal death [23]; we then tested whether the convulsive threshold to a pro-convulsive stimulus was also altered. For that purpose, animals that developed SE after pilocarpine administration were treated with gabapentin or vehicle for 4 days, and then at 21DPSE were re-exposed to sub-convulsive repetitive 10 mg/kg pilocarpine doses every 30 min. After four pilocarpine doses, all animals developed seizures; however, only 33% of the gabapentin-treated animals developed SE (limited to Racine 4) compared with 72% of the vehicle-treated animals that in all cases reached Racine 5 (Figure 9A). Figure 9A also shows that 33% of the gabapentin-treated animals reached sustained Racine 1–3 stages, while only 14% of animals of the vehicle-treated group attained this low Racine stage after re-exposure to pilocarpine. In addition, no deaths were observed in the gabapentin-treated animals while 14% of the vehicle-treated ones died after the pilocarpine-induced seizures (Figure 9A). Statistical significance of the results is shown by Fisher’s exact test, representing a typical experiment of pilocarpine re-exposure after vehicle or gabapentin treatment (Figure 9B). We conclude that early gabapentin treatment during a short time-window in the latency period produces a long-lasting reduced susceptibility to seizures.

## 3. Discussion

The cellular events that occur during the latency period that follows the IPE are still not completely understood. Although it is proposed that neuroinflammation and exacerbated synaptic plasticity occur during this period and are essential for the progression of epilepsy as a chronic disease, studies on the latency period are relatively few (see for example [5,23,39,40,41,42,43]). The supportive hypothesis underlying these studies is that a number of events which are critical for the epileptogenic process occur during the latency period that follows IPE. In addition, early therapeutic intervention during the latency period may change the natural history of the disease by preventing epileptogenesis and thus the appearance of spontaneous seizures. Several pharmacological compounds were tested during the latency period including cannabinoids [44], melatonin [45], magnesium sulfate [46] and gabapentin [23], with variable results. In fact, some treatments even showed to accelerate epileptogenesis [47,48].

The molecular and cellular changes occurring during the latency period cannot be studied in humans. Human tissue samples from TLE patients are only available from advanced, drug-resistant stages of the disease and thus the resected tissue exhibits many pathological changes not necessarily linked to epileptogenesis, but resulting from accumulated seizures, neuronal death, hippocampal sclerosis and neuroinflammation (reviewed in [49]). Thus, experimental approaches such as the lithium-pilocarpine model of TLE or the administration of pentylenetetrazole in rodents are essential to study the early events during the latency period [25,50]. We have previously observed that hippocampal neurons and piriform cortical neurons degenerate just a few days after the IPE produced by pilocarpine-induced SE [23]. In addition, reactive astrocytes appear in these areas after the IPE, but the profuse reactive gliosis disseminates along the entire brain, including distant anatomical areas [23]. Importantly, these effects have a peak around 7 to 15DPSE, before the establishing of chronic epileptic seizures. We here extended these observations and showed that the TLR4 innate immunity receptor is expressed in resident microglia, reactive astrocytes and stressed neurons from hippocampus and piriform cortex during the latency period after the IPE. The innate immunity participation in epileptogenesis has been proposed [34,51,52] and specifically TLR4 receptor expression was directly linked to epilepsy development [7,10,53,54,55]. Various experimental approaches have shown that targeting IL1β/TLR4 signaling may prevent epileptogenesis, showing disease-modifying effects [7,56]. In agreement with these reports, we observed that TLR4 expression increases during the latency period in the anatomical areas that present neurodegeneration. In glial cells, TLR4 expression was recently linked to astroglial polarization to the astroglial A1 proinflammatory-neurodegenerative phenotype, which may increase neuronal death [57,58]. Our results show that TLR4 is expressed in astrocytes and neurons from hippocampus and piriform cortex; in these areas we were also able to detect a significant number of stressed or degenerating neurons, identified by the atypical distribution of NeuN [59].

Astrocytes are known to express several pro-synaptogenic molecules including proteins, lipids, and small molecules that bind to neuronal receptors to promote synaptogenesis and regulate synaptic connectivity (reviewed in [60]). One of the best characterized astroglial-derived synaptogenic molecules is TSP-1, which specifically induces excitatory synaptogenesis [18]. Reactive astrocytes are known to re-express synaptogenic molecules that were down-regulated after development (see review in [9]). Interestingly, we here observed that reactive perivascular astrocytes express TSP-1 at early time points after the IPE, during the early latency period. Spurious excitatory synaptogenesis in the latency period is hypothesized as one of the basis of the E/I altered balance that underlies epileptogenesis, and thus we propose that reactive astrocytes induced by the IPE secrete synaptogenic molecules involved in the spurious synaptogenesis, a mechanism suggested as one of the basis of epileptogenesis.

BBB integrity is essential for normal brain function and is also a main impediment for leukocytes and blood-borne cells to enter the CNS. Endothelial cells are also important players in the neuro-vascular unit that comprises also glial cells and neurons [61]. The BBB allows a controlled entrance of certain molecules essential for the neuronal metabolism. Experimental evidence showed that BBB leakage is able to induce seizures [34,62]. As elegantly reviewed by Janigro [62], ictogenic consequences of BBB leakage are probably related to alterations in the ionic balance (specifically K^+^), deregulation of neurotransmitters/neuromodulators abundance, albumin and immune cells access to the CNS. We here observed that pilocarpine-induced IPE alters the expression of essential channels involved in the BBB permeability. Specifically, we observed overexpression of Pgp-170 (MDR-1) efflux transporter and a downregulation in the astroglial AQP4 expression during the latency period. Although it is not clear how these changes in expression correlate with alterations in the CNS homeostasis, Pgp-170 has been regarded as one of the factors inducing pharmacoresistance to AED by pumping out of the brain the active drugs [35,36]. On the other hand, AQP4 is a key molecule to maintain CNS homeostasis, specifically by keeping control of the CNS water content, and its alteration has been observed in numerous brain diseases including epilepsy (reviewed in [63]). AQP4 is expressed in astrocytes end-feet, which participate of the BBB. AQP4 down-regulation or miss-localization has been repeatedly linked to experimental epileptic seizures [64,65,66,67] and observed in human tissue from epileptic patients [68,69]. Moreover, variants of genes encoding AQP4 are associated with genetic TLE [70] and knockout mice lacking AQP4 showed increased seizure duration after in vivo cortical electrical stimulation [71]. Interestingly, we observed AQP4 downregulation in the hippocampus but also in the piriform cortex, where loss of GFAP-immunostained astrocytes was also observed. In this scenario, we could not exclude that AQP4 downregulation could be secondary to an astroglial dysfunction or malfunction provoked by the pilocarpine-induced IPE.

BBB alteration during the latency period, however, seems to involve also other players. The transference of *naïve* eGFP-BMMC to SE-exposed animals during the early latency period showed that endothelial cells are permissive for myeloid cell infiltration, especially at early time points after the IPE. In addition, we have also observed NG2+ blood-derived macrophages in the hippocampal and piriform cortex territories. This is in agreement with the recent previous work showing that vascular inflammatory mechanisms and leukocyte-endothelial adhesion can contribute to the pathogenesis of seizures and epilepsy [34,51,52]. Since it is difficult to directly show leukocyte infiltration in epileptic patients, it remains unclear how leukocyte infiltration contributes to real life seizures in humans (reviewed in [62]). Brain invasion by leukocytes and immune cells has been clearly evidenced in brain tissue resected from epileptic patients as well as in viral-triggered epilepsy [34,72]. The available data suggest that immune cell infiltration in epilepsy contributes to BBB damage and seizures [34,51,52]. In agreement with these results, we show here that IPE induces early macrophage infiltration to the brain, during the early latency period. Subsequent blood-borne macrophage interaction with resident microglia and astrocytes probably has an additional role on the microglial and astroglial activation during the latency period.

Gabapentin and pregabalin are two sister drugs that are collectively known as gabapentinoids [73]. Gabapentin was originally synthesized as an analog of the neurotransmitter γ-aminobutyric acid (GABA); however it was probed that gabapentin does not act as a GABA-mimetic nor binds to the GABA receptors. It is now recognized that gabapentinoids, including gabapentin, behave as inhibitors of α_2_δ type 1 and 2 of P/Q voltage-dependent calcium channels (VGCC) as initially described by Gee and colleagues [74]. Since VGCCs are located predominantly in presynaptic membranes, gabapentionids can control stimulus-dependent synaptic transmitter release, mainly on the excitatory synapses [74,75,76]. Despite the fact that originally gabapentin was designed as an AED, systematic reviews and meta-analysis of clinical trials have shown that gabapentin is better than placebo, but worse that other classical AED, in the treatment of refractory epilepsy [77,78]. After years of clinical trials, clinical use and pharmacovigilance, gabapentin is currently indicated for the treatment of chronic painful conditions, such as painful peripheral or central neuropathies [76,79]. Although prevailing knowledge points towards the gabapentin effect on α_2_δ1 VGCC in neuronal pre-synapses, several studies described gabapentin effects on glial cells [22,23,80,81,82], anti-inflammatory effects [83] and the ability of blocking TSP-1 synaptogenic effects [18]. Based on our previous results that show that gabapentin applied during the latency period following the IPE reduced neuronal degeneration and reactive gliosis [23], we here used a short, subacute, four-day treatment with gabapentin and observed a reduced reactive microgliosis and macrophage infiltration during the latency period. Astrocytes, on the other hand, were partially sensitive to the gabapentin effects, showing a reduction in morphological alterations at 5DPSE in the piriform cortex, but not in the stratum radiatum of CA-1 hippocampal area. Surprisingly, the four-day gabapentin treatment did not significantly change the level of the mRNA of proinflammatory cytokines IL-1β and TNFα at 5DPSE. In this scenario, we conclude that gabapentin treatment reduces reactive gliosis and macrophage infiltration during the latency period without producing sustained changes in proinflammatory cytokines. However, the four-day gabapentin treatment has persistent effects in the long term. In fact, gabapentin-treated animals showed an increased seizure threshold when they were exposed to accumulated subconvulsive doses of pilocarpine 21 days after the IPE. Gabapentin treatment in an early therapeutic window following the IPE was able to increase seizure threshold, to reduce mortality and to reduce the Racine level achieved by the animals after a pro-convulsive challenge with accumulated sub-convulsive pilocarpine administration. Separated administration of sub-convulsive doses of GABA-A antagonist pentylenetetrazole (25 days apart) has been shown to induce kindling and increase neurogenesis [84,85]. The possible gabapentin effect of pentylenetetrazole-induced kindling and neurogenesis will be the subject of future investigations. 

An obvious limitation of our study is that motor seizures were used to define the convulsive threshold and the outcome following gabapentin treatment. Video-EEG recordings showed that most of pilocarpine-treated rats, monitored by intracerebral or cortical electrodes, appear to have epileptic firing during the first week after SE [48]. Obviously, we cannot discard the gabapentin potential effects on this epileptic firing that may not be reflected into motor seizures during the initial days after pilocarpine-induced SE, but it might have consequences on the evolution of the epileptogenic process. 

In summary, we report here that a subacute four-day treatment with gabapentin initiated immediately after the IPE, produced by pilocarpine-induced SE, successfully reduced reactive gliosis, macrophage infiltration and increased convulsive threshold in the long-term. We conclude that gabapentin could be proposed as a new agent with probable activity in the epileptogenesis when applied in the latency period following IPE.

## 4. Materials and Methods

### 4.1. Ethics Statement

All procedures involving animals were conducted in accordance with our institutional guidelines, in agreement with the NIH guidelines for the Care and Use of Laboratory Animals and the principles presented in the Guidelines for the Use of Animals in Neuroscience Research by the Society for Neuroscience. The procedures were approved by the CICUAL committee of the School of Medicine, University of Buenos Aires (Res. Nr. 1278/2012). All efforts were made to minimize animal suffering and to reduce the number of animals used.

### 4.2. Materials

Antibodies were purchased from DAKO (policlonal anti-Glial Fibrillary Acidic Protein [GFAP]), Sigma (monoclonal anti-GFAP, anti-S100B), Abcam (anti-Iba-1), Millipore-Chemicon (anti-NG2), and Santa Cruz (anti-thrombospondin-1 [TSP-1]). Secondary fluorescent or biotinilated antibodies were from Jackson Immunoresearch and Sigma (St. Louis, MO, USA) respectively. Gabapentin (Neurontin) was from Pfizer (Buenos Aires, Argentina). For Quantitative RT-PCR, the Quick-RNA™ MiniPrep (ZYMO Research,#R1054, Irvine, CA, USA.) was used for RNA extraction, the High-Capacity cDNA Reverse Transcription Kit (Applied Biosystems, Foster City, CA, USA) was used for retrotranscription, and the SYBR^®^ Select Master Mix (Life Technologies, Carlsbad, CA, USA) for Real Time PCR.

### 4.3. Animals and Temporal Lobe Epilepsy Model

In this study we used adult male Wistar rats (200–250 g) from the animal breeding facility of the School of Exact and Natural Sciences (University of Buenos Aires). Animal rooms have controlled temperature, humidity and lighting conditions (20–25 °C, 60% humidity, 12 h/12 h light/dark cycle). As described previously [23], animals were subjected to the lithium-pilocarpine model of TLE. Briefly, animals were randomly separated in two experimental groups: both groups were injected intraperitoneally (IP) with 3 mEq/kg lithium chloride, and twenty hours later one group received 30 mg/kg pilocarpine (Li-Pilo group) while the other group was injected with saline solution (Control-Li group). Development of seizures was evaluated behaviorally according with the Racine scale. SE was defined when animals developed continuous seizures with a Racine score of 3 to 5, without returning to lower stages for at least 5 min. Between 40 and 60 min after pilocarpine administration, animals developed behavioral signs of SE (Racine 3–5) (SE group). A number of animals (10–20%) did not develop SE, showing only behavioral features corresponding to stages 1–2 of the Racine score, were considered as a separate experimental group (NoSE) and used in some experiments as detailed in figures. All animals received 20 mg/kg diazepam (IP) 20 min after the onset of SE to stop convulsions. SE duration was limited to 20 min to reduce animal mortality and to allow a subsequent chronic period of spontaneous seizures [25]. SE was considered as an IPE for the development of epileptogenesis. After 24 h of the SE, a gabapentin treatment was initiated: we administered daily gabapentin injections (400 mg/kg/day IP) for 4 days, or an equal volume of saline.

To carry out immunohistochemical studies, we established 3, 5 and 7 DPSE as the end-points of the experiment. Animals were anaesthetized with ketamine/xylazine (90/10 mg/kg, IP) and were perfused through the left ventricle as previously described [86]. Then, brains were cryoprotected, snap frozen and coronal 25–50 μm thick brain sections were cut using a cryostat. 

Another group of animals received 3 mEq/kg lithium chloride intraperitoneally and 20 h later were injected with 30 mg/kg pilocarpine, as described previously. SE was terminated after 20 min and 24 h later animals that developed SE were divided in two groups: the first one received 400 mg/kg/day gabapentin during 4 days, and the second one received saline. After 21DPSE, both groups were re-treated with 3 mEq/kg lithium chloride and 20 h later were exposed to subconvulsant doses of pilocarpine (10 mg/kg) repeated every 30 min up to a maximum of 4 doses. Seizure development was evaluated according to the Racine scale, and SE was arrested with diazepam as explained before.

### 4.4. Transfer of eGFP Blood Cells and Bone Marrow-Derived Progenitors

In order to evaluate the role of infiltrating cells, blood cells and bone marrow-derived progenitors were obtained from eGFP rats [Wistar-TgN (CAG-GFP)184ys] [87], and transferred to recipient animals exposed to SE, through the lateral tail vein. For the acquisition of the cells, all procedures were done under sterile conditions, and animals were deeply anesthetized with a ketamine-xylazine solution (90–10 mg/kg). BMMC fraction was obtained by Ficoll centrifugation of bone marrow extracted from the long bones of the hind limbs, as described [88]. Briefly, needle puncture was performed on the spongy bone by injecting 4 mL of DMEM supplemented with 10% fetal bovine serum. The bone marrow cell suspension was collected and slowly added to a 15 mL tube containing 2 mL of Ficoll (δ = 1.077). Centrifugation was performed at 1000 RPM for 20 min; the cell interface was collected to perform a new centrifugation at 1000 RPM for 5 min. After discarding the supernatant, the pellet was resuspended in culture medium. For the acquisition of blood cells, 5 mL of blood were obtained from the left ventricle in a heparinized syringe, transferred to a 15 mL centrifuge tube containing 10 mL of physiological solution, and centrifuged at 200 *g* for 20 min at room temperature. After discarding the plasma, the leukocyte layer was collected and the remaining red cells were lysed by incubation with 0.2% NaCl for 1 min. Osmolarity was recovered with the addition of 1 vol. of 1.6% NaCl, and the cells were centrifuged at 1000 RPM for 10 min at room temperature. The pellet obtained was re-suspended in physiological solution. In both cases, viable cells were counted with Trypan Blue in the Neubauer chamber to subsequently inject them through the lateral tail vein in the recipient animals. The volume of injection was 300 μL, and 6.6 × 10^5^ leukocytes or 15 × 10^6^–20 × 10^6^ BMMC were injected per rat. 3DPSE animals were euthanized and brains were processed for immunohistochemistry.

### 4.5. Immunohistochemistry

Brain sections of animals from all experimental groups were simultaneously processed in the free floating state as previously described [23,86]. Photographs were taken with an Olympus QColor 5 camera mounted on a Zeiss Axiophot microscope (Carl Zeiss, Germany) or with an Olympus IX-81 microscope equipped with a DP71 camera (Olympus, Tokyo, Japan). Primary antibodies dilutions were: anti-GFAP (1:1000), anti-TSP-1 (1:800), anti-NG2 (1:1000), anti-Iba-1 (1:1000), anti-S100B (1:800). Secondary Alexa 488 or 594 antibodies were used at 1:800, and nuclear counterstaining was done with 4′,6-diamidino-2-phenylindole DAPI (1 μg/mL). 

### 4.6. RNA Isolation, Reverse Transcription and Real-Time PCR 

Quantitative RT-PCR was performed as previously described [58,89]. Total left and right hippocampi were quickly dissected over ice, snap frozen in liquid nitrogen, and stored at −80 °C. Total RNA from the left hippocampus was isolated using a Bio-Gen PRO200 Homogenizer and the Quick-RNA™ MiniPrep (ZYMO Research) according to the manufacturer’s instructions, including the gDNA in column removal step. The cDNA was generated using the High-Capacity cDNA Reverse Transcription Kit (Applied Biosystems), using oligo-dT primers. Each reverse transcriptase-PCR (RT-PCR) experiment was run with negative control reactions in the absence of reverse transcriptase. qPCRs were performed using specific primers. Comparative quantification was performed by quantitative real time PCR, using the SYBR-green I fluorescence method, and ROX as passive reference dye (SYBR^®^ Select Master Mix, Life Technologies). TATA Box binding protein (TBP), Glyceraldehyde 3-phosphate dehydrogenase (GAPDH), and/or β-actin were used as housekeeping genes. Samples were run in triplicate. Primer sequences and amplification product sizes are specified in Supplementary Table S1 and detailed protocols can be requested from authors. 

### 4.7. Quantitative Studies

Experiments were repeated at least 3 times with similar results; a representative experiment is shown in the figures. A minimum of 3 animals per condition was used in each experiment. Morphometrical studies were performed as we have previously described using the NIH ImageJ software [23]. Statistical analysis was performed by one-way, two-way ANOVA, Student *t*-test or Fisher’s exact test as appropriate and post test ad hoc. Significance was set at *p* < 0.05. Data is presented as mean ± standard error of the mean (SEM).

## Supplementary Materials

The following are available online at www.mdpi.com/1424-8247/10/4/93/s1, Figure S1: TSP-1 immunostaining in control brains, Table S1: Primer sequences and expected cDNA amplification product size. Interleukin 1 beta (IL-1β), Tumor necrosis factor alpha (TNFα), TATA box binding protein (TBP), Glyceraldehyde 3-phosphate dehydrogenase (GAPDH), β-Actin.

## References

[1] Hesdorffer D.C., Beck V., Begley C.E., Bishop M.L., Cushner-Weinstein S., Holmes G.L., Shafer P.O., Sirven J.I., Austin J.K. (2013). Research implications of the Institute of Medicine Report, Epilepsy across the Spectrum: Promoting Health and Understanding. Epilepsia.

[2] Clossen B.L., Reddy D.S. (2017). Novel therapeutic approaches for disease-modification of epileptogenesis for curing epilepsy. Biochim. Biophys. Acta.

[3] Witt J.A., Hoppe C., Helmstaedter C. (2017). Neuropsychologist’s (re-)view: Resective versus ablative amygdalohippocampectomies. Epilepsy Res..

[4] Cukiert A. (2015). Vagus Nerve Stimulation for Epilepsy: An Evidence-Based Approach. Prog. Neurol. Surg..

[5] Shukla G., Prasad A.N. (2012). Natural history of temporal lobe epilepsy: Antecedents and progression. Epilepsy Res. Treat..

[6] Curia G., Lucchi C., Vinet J., Gualtieri F., Marinelli C., Torsello A., Costantino L., Biagini G. (2014). Pathophysiogenesis of mesial temporal lobe epilepsy: Is prevention of damage antiepileptogenic?. Curr. Med. Chem..

[7] Maroso M., Balosso S., Ravizza T., Liu J., Aronica E., Iyer A.M., Rossetti C., Molteni M., Casalgrandi M., Manfredi A.A. (2010). Toll-like receptor 4 and high-mobility group box-1 are involved in ictogenesis and can be targeted to reduce seizures. Nat. Med..

[8] Vezzani A., Balosso S., Maroso M., Zardoni D., Noé F., Ravizza T. (2010). ICE/caspase 1 inhibitors and IL-1beta receptor antagonists as potential herapeutics in epilepsy. Curr. Opin. Investig. Drugs.

[9] Kim S.K., Nabekura J., Koizumi S. (2017). Astrocyte-mediated synapse remodeling in the pathological brain. Glia.

[10] Shen Y., Qin H., Chen J., Mou L., He Y., Yan Y., Zhou H., Lv Y., Chen Z., Wang J. (2016). Postnatal activation of TLR4 in astrocytes promotes excitatory synaptogenesis in hippocampal neurons. J. Cell Biol..

[11] Faria L.C., Gu F., Parada I., Barres B., Luo Z.D., Prince D.A. (2017). Epileptiform activity and behavioral arrests in mice overexpressing the calcium channel subunit α2δ-1. Neurobiol. Dis..

[12] Yu C.Y., Gui W., He H.Y., Wang X.S., Zuo J., Huang L., Zhou N., Wang K., Wang Y. (2014). Neuronal and astroglial TGFβ-Smad3 signaling pathways differentially regulate dendrite growth and synaptogenesis. NeuroMol. Med..

[13] Hawrylak N., Chang F.L., Greenough W.T. (1993). Astrocytic and synaptic response to kindling in hippocampal subfield CA1. II. Synaptogenesis and astrocytic process increases to in vivo kindling. Brain Res..

[14] Niquet J., Jorquera I., Ben-Ari Y., Represa A. (1993). NCAM immunoreactivity on mossy fibers and reactive astrocytes in the hippocampus of epileptic rats. Brain Res..

[15] Lehtimäki K.A., Keränen T., Palmio J., Peltola J. (2010). Levels of IL-1beta and IL-1ra in cerebrospinal fluid of human patients after single and prolonged seizures. Neuroimmunomodulation.

[16] Li X.W., Yang F., Wang Y.G., Wang J.C., Ma L., Jiang W. (2013). Brain recruitment of dendritic cells following Li-pilocarpine induced status epilepticus in adult rats. Brain Res. Bull..

[17] Christopherson K.S., Ullian E.M., Stokes C.C., Mullowney C.E., Hell J.W., Agah A., Lawler J., Mosher D.F., Bornstein P., Barres B.A. (2005). Thrombospondins are astrocyte-secreted proteins that promote CNS synaptogenesis. Cell.

[18] Eroglu C., Allen N.J., Susman M.W., O’Rourke N.A., Park C.Y., Ozkan E., Chakraborty C., Mulinyawe S.B., Annis D.S., Huberman A.D. (2009). Gabapentin receptor alpha2delta-1 is a neuronal thrombospondin receptor responsible for excitatory CNS synaptogenesis. Cell.

[19] Celli R., Santolini I., Guiducci M., van Luijtelaar G., Parisi P., Striano P., Gradini R., Battaglia G., Ngomba R.T., Nicoletti F. (2017). The α2δ Subunit and Absence Epilepsy: Beyond Calcium Channels?. Curr. Neuropharmacol..

[20] Okada-Tsuchioka M., Segawa M., Kajitani N., Hisaoka-Nakashima K., Shibasaki C., Morinobu S., Takebayashi M. (2014). Electroconvulsive seizure induces thrombospondin-1 in the adult rat hippocampus. Prog. Neuropsychopharmacol. Biol. Psychiatry.

[21] Li H., Graber K.D., Jin S., McDonald W., Barres B.A., Prince D.A. (2012). Gabapentin decreases epileptiform discharges in a chronic model of neocortical trauma. Neurobiol. Dis..

[22] Yang J.L., Xu B., Li S.S., Zhang W.S., Xu H., Deng X.M., Zhang Y.Q. (2012). Gabapentin reduces CX3CL1 signaling and blocks spinal microglial activation in monoarthritic rats. Mol. Brain.

[23] Rossi A.R., Angelo M.F., Villarreal A., Lukin J., Ramos A.J. (2013). Gabapentin administration reduces reactive gliosis and neurodegeneration after pilocarpine-induced status epilepticus. PLoS ONE.

[24] Racine R.J. (1972). Modification of seizure activity by electrical stimulation. II. Motor seizure. Electroencephalogr. Clin. Neurophysiol..

[25] Curia G., Longo D., Biagini G., Jones R.S., Avoli M. (2008). The pilocarpine model of temporal lobe epilepsy. J. Neurosci. Methods.

[26] Gualtieri F., Marinelli C., Longo D., Pugnaghi M., Nichelli P.F., Meletti S., Biagini G. (2013). Hypoxia markers are expressed in interneurons exposed to recurrent seizures. Neuromol. Med..

[27] Lucchi C., Vinet J., Meletti S., Biagini G. (2015). Ischemic-hypoxic mechanisms leading to hippocampal dysfunction as a consequence of status epilepticus. Epilepsy Behav..

[28] Li Y., Serwanski D.R., Miralles C.P., Fiondella C.G., Loturco J.J., Rubio M.E., De Blas A.L. (2010). Synaptic and nonsynaptic localization of protocadherin-gammaC5 in the rat brain. J. Comp. Neurol..

[29] Ortiz-Masià D., Díez I., Calatayud S., Hernández C., Cosín-Roger J., Hinojosa J., Esplugues J.V., Barrachina M.D. (2012). Induction of CD36 and thrombospondin-1 in macrophages by hypoxia-inducible factor 1 and its relevance in the inflammatory process. PLoS ONE.

[30] Osada-Oka M., Ikeda T., Akiba S., Sato T. (2008). Hypoxia stimulates the autocrine regulation of migration of vascular smooth muscle cells via HIF-1alpha-dependent expression of thrombospondin-1. J. Cell. Biochem..

[31] Chen Y.C., Zhu G.Y., Wang X., Shi L., Du T.T., Liu D.F., Liu Y.Y., Jiang Y., Zhang X., Zhang J.G. (2017). Anterior thalamic nuclei deep brain stimulation reduces disruption of the blood-brain barrier, albumin extravasation, inflammation and apoptosis in kainic acid-induced epileptic rats. Neurol. Res..

[32] Michalak Z., Sano T., Engel T., Miller-Delaney S.F., Lerner-Natoli M., Henshall D.C. (2013). Spatio-temporally restricted blood-brain barrier disruption after intra-amygdala kainic acid-induced status epilepticus in mice. Epilepsy Res..

[33] Marchi N., Teng Q., Ghosh C., Fan Q., Nguyen M.T., Desai N.K., Bawa H., Rasmussen P., Masaryk T.K., Janigro D. (2010). Blood-brain barrier damage, but not parenchymal White blood cells, is a hallmark of seizure activity. Brain Res..

[34] Fabene P.F., Navarro Mora G., Martinello M., Rossi B., Merigo F., Ottoboni L., Bach S., Angiari S., Benati D., Chakir A. (2008). A role for leukocyte-endothelial adhesion mechanisms in epilepsy. Nat. Med..

[35] Lazarowski A., Ramos A.J., García-Rivello H., Brusco A., Girardi E. (2004). Neuronal and glial expression of the multidrug resistance gene product in an experimental epilepsy model. Cell. Mol. Neurobiol..

[36] Hartz A.M., Pekcec A., Soldner E.L., Zhong Y., Schlichtiger J., Bauer B. (2017). P-gp Protein Expression and Transport Activity in Rodent Seizure Models and Human Epilepsy. Mol. Pharm..

[37] Burda J.E., Sofroniew M.V. (2014). Reactive gliosis and the multicellular response to CNS damage and disease. Neuron.

[38] Benakis C., Garcia-Bonilla L., Iadecola C., Anrather J. (2015). The role of microglia and myeloid immune cells in acute cerebral ischemia. Front. Cell. Neurosci..

[39] Vizuete A.F.K., Hennemann M.M., Gonçalves C.A., de Oliveira D.L. (2017). Phase-Dependent Astroglial Alterations in Li-Pilocarpine-Induced Status Epilepticus in Young Rats. Neurochem. Res..

[40] Kim C.H. (2015). Cav3.1 T-type calcium channel modulates the epileptogenicity of hippocampal seizures in the kainic acid-induced temporal lobe epilepsy model. Brain Res..

[41] Soukupová M., Binaschi A., Falcicchia C., Zucchini S., Roncon P., Palma E., Magri E., Grandi E., Simonato M. (2014). Impairment of GABA release in the hippocampus at the time of the first spontaneous seizure in the pilocarpine model of temporal lobe epilepsy. Exp. Neurol..

[42] Soukupova M., Binaschi A., Falcicchia C., Palma E., Roncon P., Zucchini S., Simonato M. (2015). Increased extracellular levels of glutamate in the hippocampus of chronically epileptic rats. Neuroscience.

[43] Monaghan M.M., Menegola M., Vacher H., Rhodes K.J., Trimmer J.S. (2008). Altered expression and localization of hippocampal A-type potassium channel subunits in the pilocarpine-induced model of temporal lobe epilepsy. Neuroscience.

[44] Suleymanova E.M., Shangaraeva V.A., van Rijn C.M., Vinogradova L.V. (2016). The cannabinoid receptor agonist WIN55.212 reduces consequences of status epilepticus in rats. Neuroscience.

[45] Tchekalarova J., Petkova Z., Pechlivanova D., Moyanova S., Kortenska L., Mitreva R., Lozanov V., Atanasova D., Lazarov N., Stoynev A. (2013). Prophylactic treatment with melatonin after status epilepticus: Effects on epileptogenesis, neuronal damage, and behavioral changes in a kainate model of temporal lobe epilepsy. Epilepsy Behav..

[46] Oliveira L.D., Oliveira R.W., Futuro Neto H.d.A., Nakamura-Palacios E.M. (2011). The role of magnesium sulfate in prevention of seizures induced by pentylenetetrazole in rats. Arq. Neuro-Psiquiatr..

[47] Biagini G., Longo D., Baldelli E., Zoli M., Rogawski M.A., Bertazzoni G., Avoli M. (2009). Neurosteroids and epileptogenesis in the pilocarpine model: Evidence for a relationship between P450scc induction and length of the latent period. Epilepsia.

[48] Biagini G., Rustichelli C., Curia G., Vinet J., Lucchi C., Pugnaghi M., Meletti S. (2013). Neurosteroids and epileptogenesis. J. Neuroendocrinol..

[49] Majores M., Schoch S., Lie A., Becker A.J. (2007). Molecular neuropathology of temporal lobe epilepsy: Complementary approaches in animal models and human disease tissue. Epilepsia.

[50] Retchkiman I., Fischer B., Platt D., Wagner A.P. (1996). Seizure induced C-Fos mRNA in the rat brain: Comparison between young and aging animals. Neurobiol. Aging.

[51] Varvel N.H., Neher J.J., Bosch A., Wang W., Ransohoff R.M., Miller R.J., Dingledine R. (2016). Infiltrating monocytes promote brain inflammation and exacerbate neuronal damage after status epilepticus. Proc. Natl. Acad. Sci. USA.

[52] Vinet J., Vainchtein I.D., Spano C., Giordano C., Bordini D., Curia G., Dominici M., Boddeke H.W., Eggen B.J., Biagini G. (2016). Microglia are less pro-inflammatory than myeloid infiltrates in the hippocampus of mice exposed to status epilepticus. Glia.

[53] Wang F.X., Yang X.L., Ma Y.S., Wei Y.J., Yang M.H., Chen X., Chen B., He Q., Yang Q.W., Yang H. (2017). TRIF contributes to epileptogenesis in temporal lobe epilepsy during TLR4 activation. Brain Behav. Immun..

[54] Yang W., Li J., Shang Y., Zhao L., Wang M., Shi J., Li S. (2017). HMGB1-TLR4 Axis Plays a Regulatory Role in the Pathogenesis of Mesial Temporal Lobe Epilepsy in Immature Rat Model and Children via the p38MAPK Signaling Pathway. Neurochem. Res..

[55] Pernhorst K., Herms S., Hoffmann P., Cichon S., Schulz H., Sander T., Schoch S., Becker A.J., Grote A. (2013). TLR4, ATF-3 and IL8 inflammation mediator expression correlates with seizure frequency in human epileptic brain tissue. Seizure.

[56] Iori V., Iyer A.M., Ravizza T., Beltrame L., Paracchini L., Marchini S., Cerovic M., Hill C., Ferrari M., Zucchetti M. (2017). Blockade of the IL-1R1/TLR4 pathway mediates disease-modification therapeutic effects in a model of acquired epilepsy. Neurobiol. Dis..

[57] Liddelow S.A., Guttenplan K.A., Clarke L.E., Bennett F.C., Bohlen C.J., Schirmer L., Bennett M.L., Münch A.E., Chung W.S., Peterson T.C. (2017). Neurotoxic reactive astrocytes are induced by activated microglia. Nature.

[58] Rosciszewski G., Cadena V., Murta V., Lukin J., Villarreal A., Roger T., Ramos A.J. (2017). Toll-Like Receptor 4 (TLR4) and Triggering Receptor Expressed on Myeloid Cells-2 (TREM-2) Activation Balance Astrocyte Polarization into a Proinflammatory Phenotype. Mol. Neurobiol..

[59] Robertson C.L., Puskar A., Hoffman G.E., Murphy A.Z., Saraswati M., Fiskum G. (2006). Physiologic progesterone reduces mitochondrial dysfunction and hippocampal cell loss after traumatic brain injury in female rats. Exp. Neurol..

[60] Baldwin K.T., Eroglu C. (2017). Molecular mechanisms of astrocyte-induced synaptogenesis. Curr. Opin. Neurobiol..

[61] Del Zoppo G.J. (2009). Inflammation and the neurovascular unit in the setting of focal cerebral ischemia. Neuroscience.

[62] Janigro D. (2012). Are you in or out? Leukocyte, ion, and neurotransmitter permeability across the epileptic blood-brain barrier. Epilepsia.

[63] Hubbard J.A., Szu J.I., Binder D.K. (2017). The role of aquaporin-4 in synaptic plasticity, memory and disease. Brain Res. Bull..

[64] Hubbard J.A., Szu J.I., Yonan J.M., Binder D.K. (2016). Regulation of astrocyte glutamate transporter-1 (GLT1) and aquaporin-4 (AQP4) expression in a model of epilepsy. Exp. Neurol..

[65] Alvestad S., Hammer J., Hoddevik E.H., Skare Ø., Sonnewald U., Amiry-Moghaddam M., Ottersen O.P. (2013). Mislocalization of AQP4 precedes chronic seizures in the kainate model of temporal lobe epilepsy. Epilepsy Res..

[66] Lee D.J., Hsu M.S., Seldin M.M., Arellano J.L., Binder D.K. (2012). Decreased expression of the glial water channel aquaporin-4 in the intrahippocampal kainic acid model of epileptogenesis. Exp. Neurol..

[67] Kim J.E., Yeo S.I., Ryu H.J., Kim M.J., Kim D.S., Jo S.M., Kang T.C. (2010). Astroglial loss and edema formation in the rat piriform cortex and hippocampus following pilocarpine-induced status epilepticus. J. Comp. Neurol..

[68] Medici V., Frassoni C., Tassi L., Spreafico R., Garbelli R. (2011). Aquaporin 4 expression in control and epileptic human cerebral cortex. Brain Res..

[69] Eid T., Lee T.S., Thomas M.J., Amiry-Moghaddam M., Bjørnsen L.P., Spencer D.D., Agre P., Ottersen O.P., de Lanerolle N.C. (2005). Loss of perivascular aquaporin 4 may underlie deficient water and K^+^ homeostasis in the human epileptogenic hippocampus. Proc. Natl. Acad. Sci. USA.

[70] Heuser K., Nagelhus E.A., Taubøll E., Indahl U., Berg P.R., Lien S., Nakken S., Gjerstad L., Ottersen O.P. (2010). Variants of the genes encoding AQP4 and Kir4.1 are associated with subgroups of patients with temporal lobe epilepsy. Epilepsy Res..

[71] Binder D.K., Yao X., Verkman A.S., Manley G.T. (2006). Increased seizure duration in mice lacking aquaporin-4 water channels. Acta Neurochir. Suppl..

[72] Vezzani A., Granata T. (2005). Brain inflammation in epilepsy: Experimental and clinical evidence. Epilepsia.

[73] Rogawski M.A., Bazil C.W. (2008). New molecular targets for antiepileptic drugs:alpha(2)delta, SV2A, and K(v)7/KCNQ/M potassium channels. Curr. Neurol. Neurosci. Rep..

[74] Gee N.S., Brown J.P., Dissanayake V.U., Offord J., Thurlow R., Woodruff G.N. (1996). The novel anticonvulsant drug, gabapentin (Neurontin), binds to the alpha2delta subunit of a calcium channel. J. Biol. Chem..

[75] Bonnet U., Scherbaum N. (2017). How addictive are gabapentin and pregabalin? A systematic review. Eur. Neuropsychopharmacol..

[76] Calandre E.P., Rico-Villademoros F., Slim M. (2016). Alpha(2)delta ligands, gabapentin, pregabalin and mirogabalin: A review of their clinical pharmacology and therapeutic use. Expert Rev. Neurother..

[77] Al-Bachari S., Pulman J., Hutton J.L., Marson A.G. (2013). Gabapentin add-on for drug-resistant partial epilepsy. Cochrane Database Syst. Rev..

[78] Costa J., Fareleira F., Ascenção R., Borges M., Sampaio C., Vaz-Carneiro A. (2011). Clinical comparability of the new antiepileptic drugs in refractory partial epilepsy: A systematic review and meta-analysis. Epilepsia.

[79] Shanthanna H., Gilron I., Rajarathinam M., AlAmri R., Kamath S., Thabane L., Devereaux P.J., Bhandari M. (2017). Benefits and safety of gabapentinoids in chronic low back pain: A systematic review and meta-analysis of randomized controlled trials. PLoS Med..

[80] Rosa A.S., Freitas M.F., Rocha I.R., Chacur M. (2017). Gabapentin decreases microglial cells and reverses bilateral hyperalgesia and allodynia in rats with chronic myositis. Eur. J. Pharmacol..

[81] Reda H.M., Zaitone S.A., Moustafa Y.M. (2016). Effect of levetiracetam versus gabapentin on peripheral neuropathy and sciatic degeneration in streptozotocin-diabetic mice: Influence on spinal microglia and astrocytes. Eur. J. Pharmacol..

[82] Wodarski R., Clark A.K., Grist J., Marchand F., Malcangio M. (2009). Gabapentin reverses microglial activation in the spinal cord of streptozotocin-induced diabetic rats. Eur. J. Pain.

[83] Dias J.M., de Brito T.V., de Aguiar Magalhães D., da Silva Santos P.W., Batista J.A., do Nascimento Dias E.G., de Barros Fernandes H., Damasceno S.R., Silva R.O., Aragão K.S. (2014). Gabapentin, a synthetic analogue of gamma aminobutyric acid, reverses systemic acute inflammation and oxidative stress in mice. Inflammation.

[84] Schmoll H., Badan I., Grecksch G., Walker L., Kessler C., Popa-Wagner A. (2003). Kindling status in sprague-dawley rats induced by pentylenetetrazole: Involvement of a critical development period. Am. J. Pathol..

[85] Buga A.M., Vintilescu R., Balseanu A.T., Pop O.T., Streba C., Toescu E., Popa-Wagner A. (2012). Repeated PTZ treatment at 25-day intervals leads to a highly efficient accumulation of doublecortin in the dorsal hippocampus of rats. PLoS ONE.

[86] Aviles-Reyes R.X., Angelo M.F., Villarreal A., Rios H., Lazarowski A., Ramos A.J. (2010). Intermittent hypoxia during sleep induces reactive gliosis and limited neuronal death in rats: Implications for sleep apnea. J. Neurochem..

[87] Mothe A.J., Kulbatski I., van Bendegem R.L., Lee L., Kobayashi E., Keating A., Tator C.H. (2005). Analysis of green fluorescent protein expression in transgenic rats for tracking transplanted neural stem/progenitor cells. J. Histochem. Cytochem..

[88] Villarreal A., Rosciszewski G., Murta V., Cadena V., Usach V., Dodes-Traian M.M., Setton-Avruj P., Barbeito L.H., Ramos A.J. (2016). Isolation and Characterization of Ischemia-Derived Astrocytes (IDAs) with Ability to Transactivate Quiescent Astrocytes. Front. Cell. Neurosci..

[89] Murta V., Farías M.I., Pitossi F.J., Ferrari C.C. (2015). Chronic systemic IL-1β exacerbates central neuroinflammation independently of the blood-brain barrier integrity. J. Neuroimmunol..

