# Supplementary material for: Early Gabapentin Treatment during the Latency Period Increases Convulsive Threshold, Reduces Microglial Activation and Macrophage Infiltration in the Lithium-Pilocarpine Model of Epilepsy
Source: Pharmaceuticals (Basel). 2017 Nov 28;10(4):93. doi: 10.3390/ph10040093 (PMC5748648; doi:10.3390/ph10040093)
Supplement: Supplementary file 1 [file pharmaceuticals-10-00093-s001.docx]

**Supplementary Figure 1**: TSP-1 immunostaining in control brains


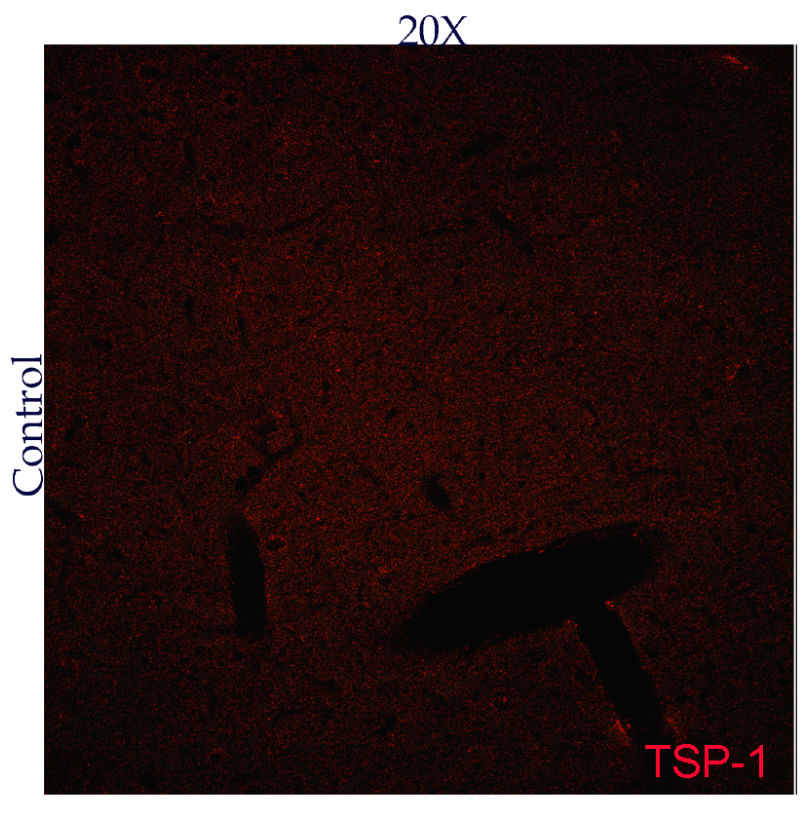


**Supplementary Table 1**. Primer sequences and expected cDNA amplification product size. Interleukin 1 beta (IL-1β), Tumor necrosis factor alpha (TNFα), TATA box binding protein (TBP), Glyceraldehyde 3-phosphate dehydrogenase (GAPDH), β-Actin.

| Gene | Primer | Sequence (5´ to 3´) | | Amplification product size |
| --- | --- | --- | --- | --- |
| IL-1β | F | TCC ATG AGC TTT GTA CAA GG | 237 bp | |
|  | R | GGT GCT GAT GTA CCA GTT GG |  |  |
| TNF-α | F | GTA GCC CAC GTC GTA GCA AA | 217 bp | |
|  | R | AAA TGG CAA ATC GGC TGA CG |  |  |
| TBP | F | ACC GTG AAT CTT GGC TGT AA | 114 bp | |
|  | R | CCT TCA TTG ACC TCA ACT ACATGG |  |  |
| GAPDH | F | AGT CTT CTG GGT GGC AGT GAT GG | 455 bp | |
|  | R | AGT CTT CTG GGT GGC AGT GAT GG |  |  |
| β-Actin | F | CAC CAC TTT CTA CAA TGA GC | 323 bp | |
|  | R | CGG TCA GGA TCT TCA TGA GG |  |  |
